# lncRNA FGD5-AS1 is required for gastric cancer proliferation by inhibiting cell senescence and ROS production via stabilizing YBX1

**DOI:** 10.1186/s13046-024-03103-x

**Published:** 2024-07-05

**Authors:** Shanshan Qin, Yue Liu, Xiangang Zhang, Pan Huang, Lingyun Xia, Weidong Leng, Dandan Li

**Affiliations:** 1 Department of Stomatology, Taihe Hospital and Hubei Key Laboratory of Embryonic Stem Cell Research, School of Basic Medical Sciences, Hubei University of Medicine, Shiyan, Hubei 442000 China; 2 Laboratory of Tumor Biology, Academy of Bio-Medicine Research, Hubei University of Medicine, Shiyan, Hubei P.R. China; 3 Shiyan Key Laboratory of Natural Medicine Nanoformulation Research, Hubei University of Medicine, Shiyan, Hubei 442000 China

**Keywords:** Cell senescence, ROS, Transcriptional regulation, RNA-protein interaction, Gastric cancer

## Abstract

**Background:**

The vast majority of lncRNAs have low expression abundance, which greatly limits their functional range and impact. As a high expression abundance lncRNA, FGD5-AS1’s non-ceRNA biological function in cancer is unclear.

**Methods:**

RNA-seq studies and chromatin immunoprecipitation (Chip) assays were performed to identify ZEB1-regulated lncRNAs. RNA sequencing, RNA pulldown, RNA Immunoprecipitation assays, and rescue assays were conducted to explore the molecular mechanisms of FGD5-AS1 in GC.

**Results:**

As one of the most abundant lncRNAs in cells, FGD5-AS1 has been shown to be transcriptionally activated by ZEB1, thus closely related to epithelial-mesenchymal transition (EMT) signaling. Clinical analysis showed that FGD5-AS1 overexpression was clinically associated with lymph node metastasis, and predicted poor survival in GC. Loss-of-function studies confirmed that FGD5-AS1 knockdown inhibited GC proliferation and induced cisplatin chemosensibility, cell senescence, and DNA damage in GC cells. Mechanismically, FGD5-AS1 is a YBX1-binding lncRNA due to its mRNA contains three adjacent structural motifs (UAAUCCCA, ACCAGCCU, and CAGUGAGC) that can be recognized and bound by YBX1. And this RNA-protein interaction prolonged the half-life of the YBX1 protein in GC. Additionally, a rescue assay showed that FGD5-AS1 promotes GC by repressing cell senescence and ROS production via YBX1.

**Conclusion:**

FGD5-AS1 is a cellular high-abundant lncRNA that is transcriptionally regulated by ZEB1. FGD5-AS1 overexpression promoted GC progression by inhibiting cell senescence and ROS production through binding and stabilizing the YBX1 protein.

**Supplementary Information:**

The online version contains supplementary material available at 10.1186/s13046-024-03103-x.

## Highlights

FGD5-AS1 is an EMT-related, cellularly abundant lncRNA. The expression of FGD5-AS1 is directly regulated by the transcription factor ZEB1.

FGD5-AS1 overexpression was clinically associated with lymph node metastasis and a poor prognosis in GC.

Knockdown of FGD5-AS1 causes senescence-associated secretory phenotypes, DNA damage, cisplatin chemosensibility, and growth inhibition in GC cells.

FGD5-AS1 prolongs the half-life of the YBX1 protein by RNA-protein interaction. The UAAUCCCA, ACCAGCCU, and CAGUGAGC linear 8-mer motifs in the FGD5-AS1 transcript mediate the interaction between YBX1 and FGD5-AS1.

FGD5-AS1 promotes GC proliferation by inhibiting cell senescence and ROS production in a YBX1-dependent manner.

**Supplementary Information:**

The online version contains supplementary material available at 10.1186/s13046-024-03103-x.

## Introduction

Gastric cancer (GC) is one of the cancers with the highest incidence rate and mortality in the world [1, 2]. The general therapy for GC patients is surgical resection accompanied by radiotherapy and chemotherapy; however the overall survival for GC patients remains relatively unsatisfactory [3]. Due to the relatively low early diagnosis rate, many GC patients were diagnosed at an advanced stage with limited therapeutic options [4]. Meanwhile, the leading cause of death for advanced GC patients is metastasis. Thus, revealing the causes of tumor metastasis or progression can help us develop better anticancer targets.

Tumorigenesis and cancer metastasis are inevitably accompanied by abnormal expression of many genes, including protein coding genes and non-coding RNAs [5, 6]. Among those dysregulated genes in cancer, some are tumor driver genes, including oncogenes and tumor suppressor genes, while others are passenger genes. It is generally believed that aberrantly expressed tumor driver genes are involved in facilitating carcinogenesis, while abnormal expression of passenger genes is essentially a byproduct or result of carcinogenesis [7]. Therefore, targeting tumor driver genes is more beneficial for cancer treatment.

Long non-coding RNAs (lncRNAs) are a group of cellular endogenous non-coding RNAs that are transcribed by RNA polymerase II. Increasing evidence has confirmed the important role of lncRNA in tumor occurrence and metastasis, indicating that lncRNA can also be a tumor driver gene. Similar to the protein-coding genes, the uncontrolled transcription regulation process is an important cause of abnormal lncRNA expression. The aberrantly expressed transcription factors should be at least partially responsible for the dysregulation of lncRNAs.

Epithelial mesenchymal transition (EMT) is recognized as a key biological process in cancer metastasis. Indubitably, the EMT-related transcription factors (EMT-TFs) could drive cancer metastasis through transcriptionally regulating downstream lncRNAs. However, due to the low abundance of lncRNA expression (which may only have a few to dozens of transcripts in a single cell), the extent and scope of lncRNA performing biological functions will inevitably be greatly compromised [8]. Although the overwhelming majority of lncRNAs are lowly expressed in cells, there are still a few lncRNAs that maintain high abundance in cells, such as MALAT1, NEAT1, and NORAD. Correspondingly, these lncRNAs with high expression abundance have indeed been confirmed to play extremely broad and profound biological functions [9–11]. Therefore, lncRNAs with high expression abundance should theoretically be more worthy of attention.

As a member of the zinc finger transcription factor family, ZEB1 is known to be one of the EMT-TFs [12, 13]. Accumulating evidence has shown that transcription factor ZEB1 is widely overexpressed in cancers, including GC [14–17]. In addition to protein-coding genes, ZEB1 is bound to promote tumor metastasis by regulating downstream lncRNAs. However, little is currently known about which lncRNAs are transcriptionally regulated by ZEB1 in GC. In this study, we performed RNA sequencing studies to uncover the downstream target lncRNAs of ZEB1. Notably, we identified FGD5-AS1 as a high-abundance lncRNA that was transcriptionally activated by ZEB1 in GC. Although many reports have shown that FGD5-AS1 plays an oncogenic role in various tumors, these reports simply focus on the competitive endogenous RNA (ceRNA) function of FGD5-AS1. The non-ceRNA function of FGD5-AS1 is currently unknown. In this study, we identified FGD5-AS1 as a YBX1-binding lncRNA since its mRNA contains 3 linear 8-mer YBX1-binding motifs (UAAUCCCA, ACCAGCCU, and CAGUGAGC). Our finding highlighted that lncRNA FGD5-AS1 promotes GC progression by repressing cell senescence via binding and stabilizing the YBX1 protein.

## Results

### Exploration of lncRNAs regulated by transcription factor ZEB1 in GC

**Fig. 1:**
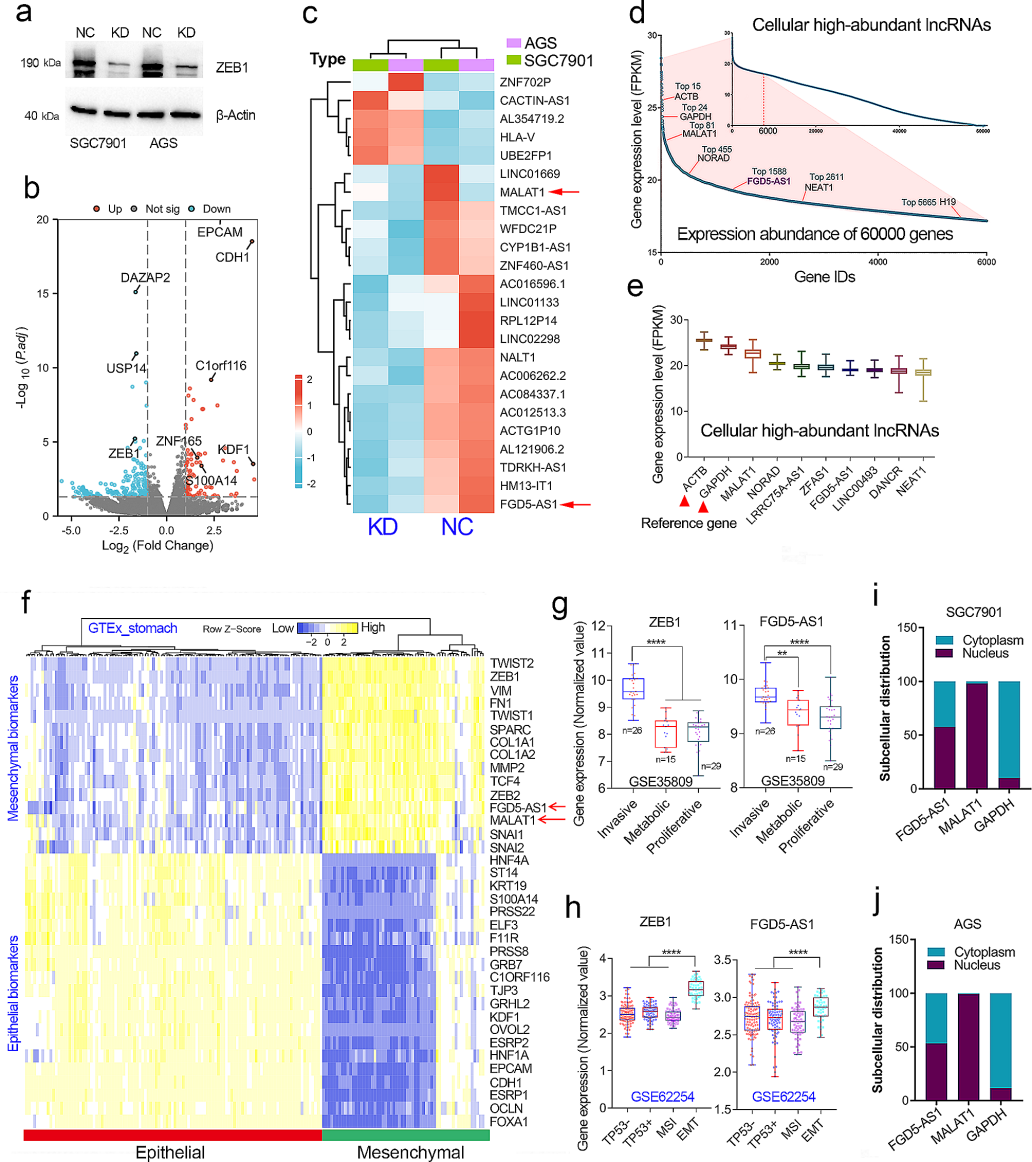
FGD5-AS1 is an EMT-related, highly abundant lncRNA in GC. (**a**) The knockdown efficiency of ZEB1 was examined by western blotting assay in the ZEB1-depleted GC cell lines. (**b**) RNA sequencing studies were conducted in the ZEB1-depleted GC cell lines. As expected, the expression of the classic epithelial biomarkers was greatly increased. (**c**) The top 24 differentially expressed lncRNAs after ZEB knockdown were shown in the heat map. (**d**) According to the normalized expression level (FPKM value) of each gene (including protein-coding genes and lncRNAs), FGD5-AS1 was a highly abundant lncRNA in GC. (**e**) FGD5-AS1 is one of 10 lncRNAs with the highest expression in GC. (**f**) MALAT1 and FGD5-AS1 were both EMT-related lncRNAs that are highly co-expressed with mesenchymal biomarkers in GC. (**g**) Both ZEB1 and FGD5-AS1 were highly expressed in the invasive subtype (also known as the mesenchymal phenotype) in the GSE35809 cohort. (**h**) Both ZEB1 and FGD5-AS1 were highly expressed in the EMT subtype GC patients of the GSE62254 cohort. (**i**, **j**) The subcellular distribution of lncRNA FGD5-AS1 was examined by nuclear-cytoplasmic separation assay in the SGC7901 and AGS cell lines

Clinical analysis showed that ZEB1 overexpression is closely related to malignant progression, lymph node metastasis, and the poor prognosis of GC patients (Fig S1a-h). As a transcription factor, ZEB1 might drive tumor metastasis by regulating downstream lncRNAs. Therefore, we have decided to explore the potent lncRNAs regulated by ZEB1 in GC. Firstly, we obtained ZEB-depleted GC cell lines by the RNA interference method (Fig. 1a). RNA-sequencing studies (GSE214471) indicated that many epithelial biomarkers (EPCAM, CDH1, C1ORF116, S100A14, ZNF165, and KDF1) were greatly upregulated (*p* < 0.001), suggesting that ZEB1 expression has been successfully knocked down in GC cell lines (Fig. 1b). Then, the top 24 lncRNAs with the most significant difference in expression after ZEB1 knockdown was displayed in the heat map (Fig. 1c). Among them, MALAT1 and FGD5-AS1 were confirmed to be the cellular high-abundant lncRNAs (Fig. 1d, e). Molecular clustering analysis using the RNA-seq data in the GTEx cohort showed that MALAT1 and FGD5-AS1 were EMT-related lncRNAs and highly co-expressed with mesenchymal biomarkers in normal stomach tissues (Fig. 1f). Lei et al. have classified GC patients in the GSE35809 cohort (*N* = 70) into 3 subtypes, including proliferative, metabolic, and mesenchymal [18]. Cristescu et al. have reported that GC could be further divided into four subtypes, including MSS/TP5−, MSS/TP53+, MSI, and MSS/EMT subtypes [19]. Expression analysis in the two independent GC cohorts also showed that FGD5-AS1 and ZEB1 were highly expressed in the EMT subtype or invasive subtype of GC tissues (Fig. 1g, h). These results strongly implied that FGD5-AS1 was a cellular high-abundant lncRNA closely related to EMT or metastasis in GC. Interestingly, unlike the nucleus-localized abundant lncRNA MALAT1, lncRNA FGD5-AS1 was distributed in both the nucleus and cytoplasm of GC cells (Fig. 1i, j).

### LncRNA FGD5-AS1 was transcriptionally activated by the EMT-TF ZEB1

**Fig. 2:**
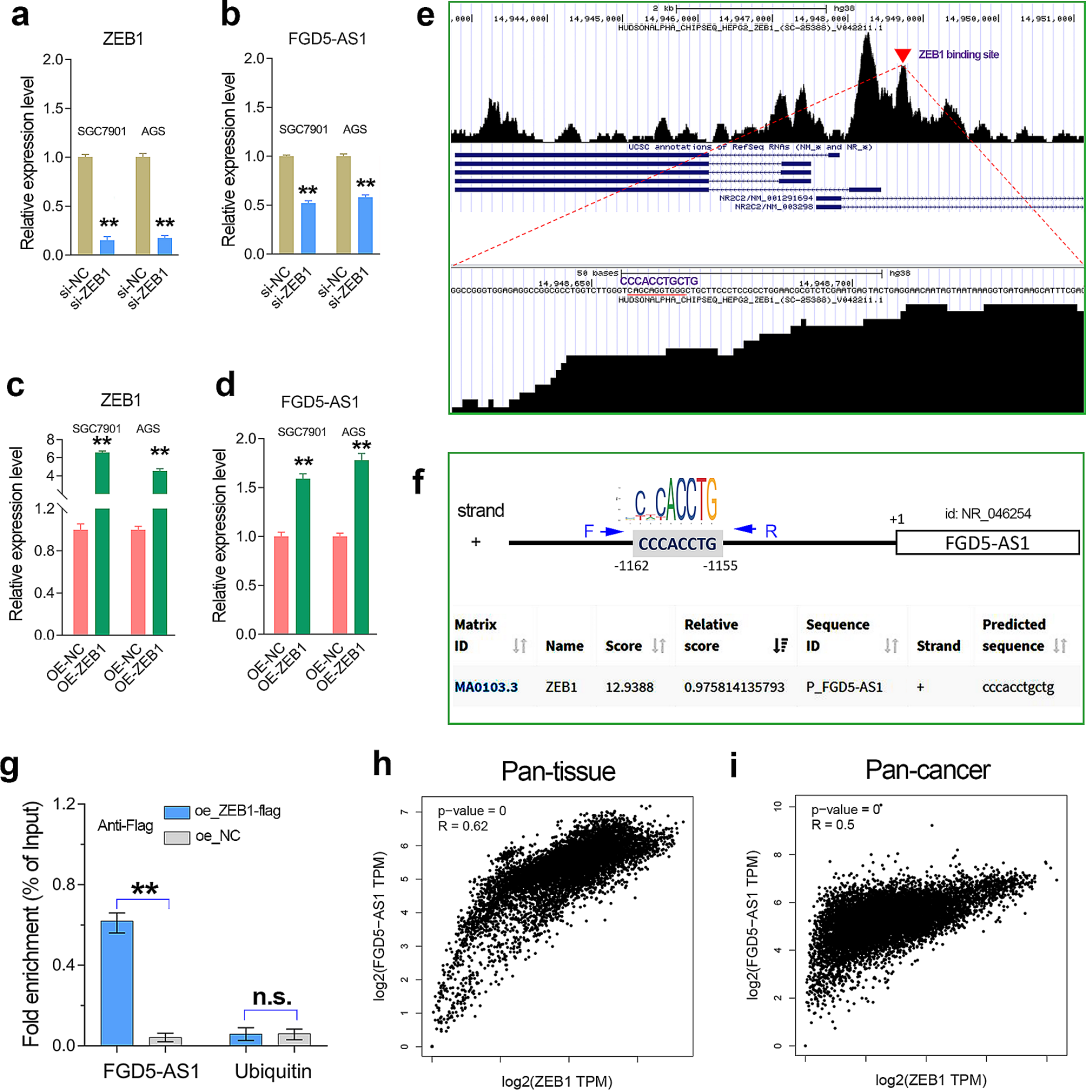
LncRNA FGD5-AS1 was transcriptionally induced by ZEB1 in GC. (**a**) The knockdown efficiency of ZEB1 in the GC cell lines was verified by the qRT-PCR analysis. (**b**) The FGD5-AS1 expression was detected in the GC cell lines with or without ZEB1 knockdown. (**c**) The overexpression efficiency of ZEB1 in GC cell lines was verified by qRT-PCR assay. (**d**) Overexpression of ZEB1 significantly increased the expression level of FGD5-AS1 in the GC cell lines. (**e**) The chip-seq analysis using the Cistrome web tool confirmed that ZEB1 can bind to the promoter of FGD5-AS1 by recognizing the “CCCA.CCTGCTG” motif in the HepG2 cell line. (**f**) Promoter analysis showed that the promoter DNA of the FGD5-AS1 gene contains a conserved ZEB1 binding motif. (**g**) The chip-qPCR analysis confirmed that ZEB1 can directly bind to the promoter of FGD5-AS1 in the AGS cells transfected with ZEB1 overexpression plasmids. (**h**, **i**) Transcription factor ZEB1 was highly co-expressed with FGD5-AS1 in pan-tissue (GTEx cohort) and pan-cancer (TCGA cohort), indicating the transcriptional regulation of FGD5-AS1 by ZEB1 may be conserved in human cells and tissues. **, *P* < 0.01

To further verify whether FGD5-AS1 can be regulated by ZEB1, a real-time quantitation RT-PCR assay was conducted. The results showed that ZEB1 knockdown significantly reduced the expression of FGD5-AS1 in the GC cell lines (Fig. 2a, b). On the contrary, stable overexpression of ZEB1 significantly upregulated the mRNA level of FGD5-AS1 in GC cell lines (Fig. 2c, d). These results implied that the EMT-TF ZEB1 positively regulated the expression of FGD5-AS1 in GC.

As a transcription factor, ZEB1 is likely to regulate the expression of FGD5-AS1 by binding to the promoter. To verify this possibility, chip-seq analysis was conducted using the Cistrome web tool. The results showed that there is a significant binding between ZEB1 and the FGD5-AS1 promoter (Fig. 2e). Moreover, promoter analysis using the JASPAR web tool showed that the central region of a ZEB1-binding peak (red arrow) contains a conserved ZEB1-recognized binding motif, “CCCACCTG” (Fig. 2f). This means that ZEB1 may directly regulate FGD5-AS1 expression at the transcriptional level. Thus, we conducted a chip-qPCR assay in the AGS cells transfected with ZEB1-flag plasmids. The results confirmed that transcription factor ZEB1 directly bound to the promoter of the FGD5-AS1 gene (Fig. 2g), indicating that FGD5-AS1 was a downstream target lncRNA of ZEB1 in GC. Additionally, considering the high co-expression between ZEB1 and FGD5-AS1 in pan-cancer and pan-tissue (*p* < 0.0001, Fig. 2h, i), we implied that the transcriptional regulation of FGD5-AS1 by ZEB1 may be widespread in human tissues and cancers.

### FGD5-AS1 overexpression was associated with metastasis and a poor prognosis in GC

**Fig. 3:**
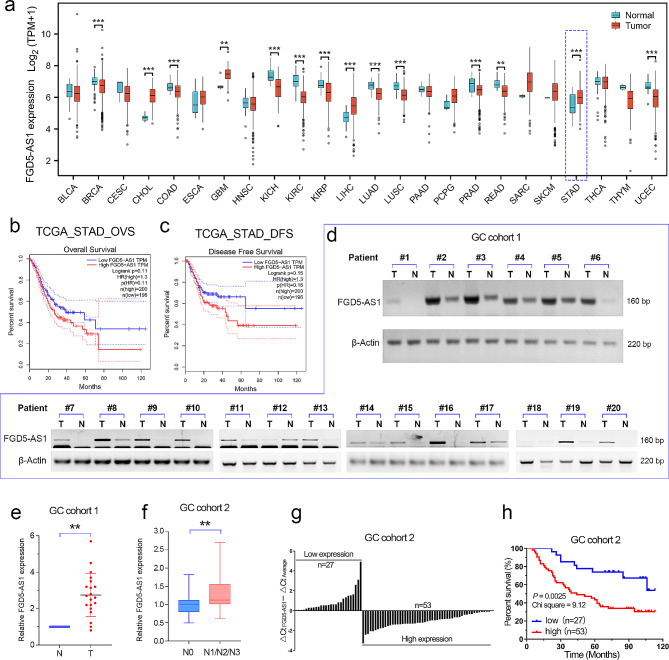
FGD5-AS1 overexpression predicted a poor prognosis in GC. (**a**) Pan-cancer analysis showed that FGD5-AS1 is frequently dysregulated in cancers and is overexpressed in stomach cancer. (**b**, **c**) Survival analysis using the GEPIA web tool showed that FGD5-AS1 overexpression predicted poor overall survival and disease-free survival in GC. (**d**, **e**) The expression level of FGD5-AS1 was evaluated in GC cohort 1 (*n* = 20) using RT-PCR analysis. The results showed that FGD5-AS1 expression was significantly increased in the GC tissues, compared to the normal adjacent tissues. (**f**) The relative expression level of FGD5-AS1 was evaluated in GC cohort 2 (*n* = 80). Clinical analysis showed that FGD5-AS1 was positively correlated with lymph node metastasis in GC cohort 2. (**g**) According to the relative FGD5-AS1 expression level, the GC patients in the GC cohort 2 were divided into two groups, FGD5-AS1_high group and the FGD5-AS1_low group. (**h**) Prognostic analysis showed that GC patients in the FGD5-AS1_high group had a shorter overall survival than the patients in the FGD5-AS1_low group. **, *P* < 0.01

As a cellular high-abundant lncRNA, FGD5-AS1 is frequently dysregulated in cancers (Fig. 3a). According to the pan-cancer analysis of the TCGA cohort, FGD5-AS1 was significantly overexpressed in GC. On the other hand, we further analyzed the prognostic value of lncRNA FGD5-AS1 in GC. Consistently, prognostic analysis showed that GC patients with relatively high FGD5-AS1 expression possessed shorter overall survival and disease-free survival in GC (Fig. 3b, c).

Furthermore, we validated the expression pattern of lncRNA FGD5-AS1 in our own cohorts by qRT-PCR assay. The GC cohort 1 containing twenty paired GC tissues and their corresponding adjacent tissues were collected to further validate the expression features of FGD5-AS1. The results showed that FGD5-AS1 was significantly overexpressed in GC cohort 1 (Fig. 3d, e). Next, we evaluated FGD5-AS1 expression by qRT-PCR analysis in the GC cohort 2, which contains detailed clinical, pathological, and prognostic information for 80 gastric cancer patients. The results showed that FGD5-AS1 was significantly correlated with lymph node metastasis (Fig. 3f). In addition, according to the relative expression level of FGD5-AS1, the 80 patients in GC cohort 2 were divided into the high FGD5-AS1 expression group (*n* = 53) and the low FGD5-AS1 expression group (*n* = 27, Fig. 3g). Prognostic analysis showed that GC patients with relatively higher FGD5-AS1 expression possessed a shorter overall survival time (*p* < 0.01, Fig. 3h).

### FGD5-AS1 knockdown inhibits GC proliferation but promotes GC cell chemosensibility and PANoptosis

**Fig. 4:**
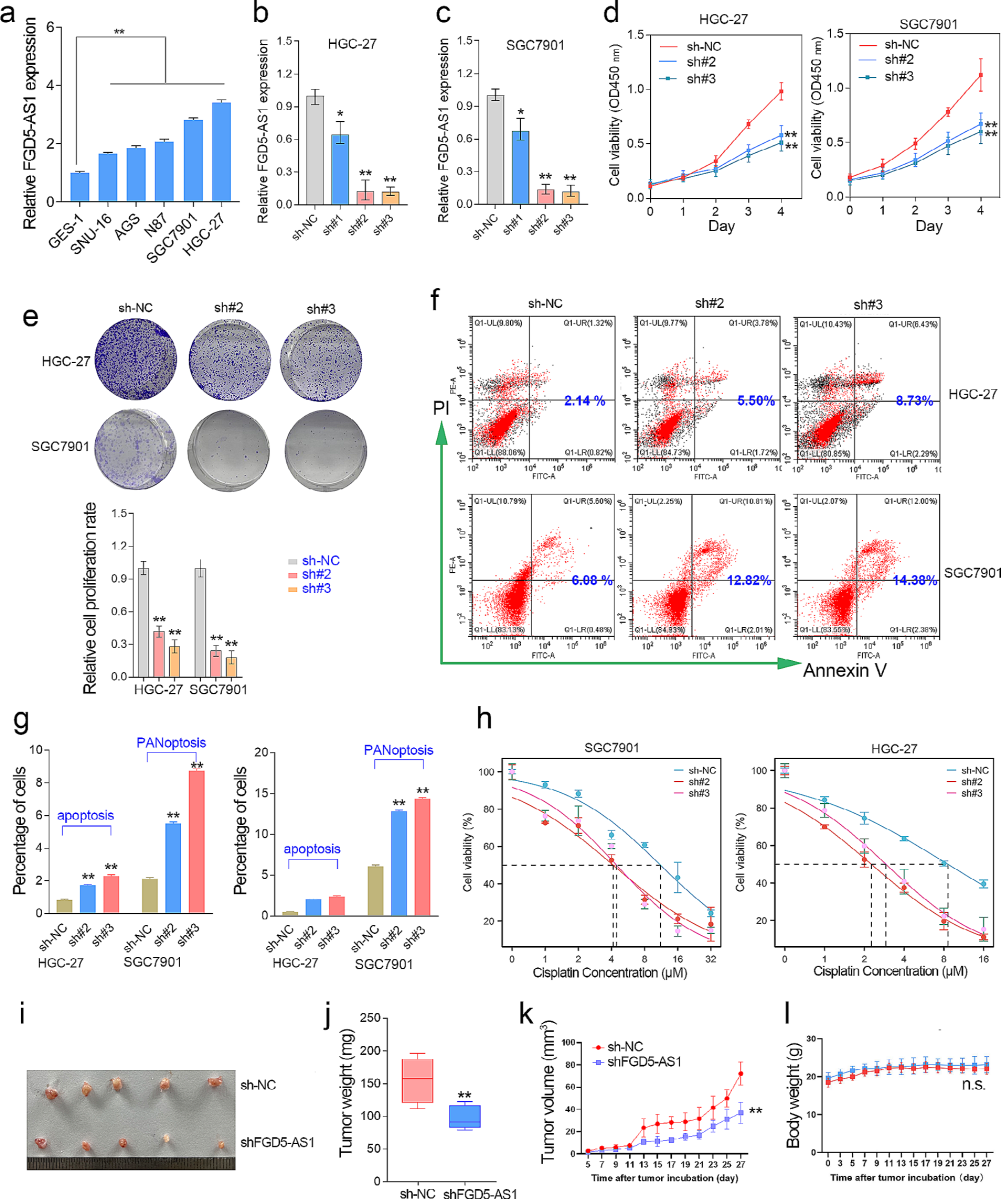
FGD5-AS1 knockdown inhibits cell proliferation and cisplatin chemoresistance in GC. (**a**) The relative expression level of FGD5-AS1 was examined in the five GC cell lines and the normal gastric cell line GES-1. (**b**, **c**) FGD5-AS1 was successfully silenced in the HGC-27 and SGC7901 cell lines. (**d**) The CCK-8 assays confirmed that FGD5-AS1 depletion significantly inhibited cell growth in the GC cell lines. (**e**) The cell colony formation assay was performed in the GC cell lines with or without FGD5-AS1 knockdown. (**f**, **g**) The cell apoptosis experiments showed that FGD5-AS1 knockdown significantly increased the number of apoptotic and PANoptotic cells in the HGC-27 and SGC7901 cell lines. (**h**) The CCK-8 assays confirmed that FGD5-AS1 depletion significantly decreased the chemoresistance of cisplatin in the GC cell lines. (**i**, **j**) After 30 days of subcutaneous tumor bearing, nude mice were euthanized. The subcutaneous xenograft tumor in nude mice was taken out and weighed. (**k**) Tumor volumes for the indicated day after injecting shNC and shFGD5-AS1 cells into nude mice. Data represent mean tumor volumes ± SEM. (**l**) The body weight of nude mice for the indicated day after injecting shNC and shFGD5-AS1 cells into nude mice. The data represent mean tumor volumes ± SEM. **, *P* < 0.01

Based on our above results, lncRNA FGD5-AS1 may play an oncogenic role in GC. Thus, we aimed to validate the oncogenic effects of FGD5-AS1 in vitro and in vivo. Admittedly, it is difficult to achieve gain-of-function studies since FGD5-AS1 is a high-abundant lncRNA that has different transcript types due to alternative splicing. Thus, a loss-of-function study was conducted using lentivirus transfection in the GC cell lines with relatively high FGD5-AS1 expression (Fig. 4a-c). Cell proliferation assay confirmed that knockdown of FGD5-AS1 significantly decreased cell growth in GC cell lines (Fig. 4d). The colony formation assay showed that knockdown of FGD5-AS1 obviously inhibited GC cell proliferation (Fig. 4e). The cell apoptosis assay showed that depletion of FGD5-AS1 significantly induced apoptosis and PANoptosis in GC cells (Fig. 4f, g). Moreover, FGD5-AS1 knockdown greatly inhibited the cisplatin chemoresistance of GC cells (Fig. 4h). Additionally, we evaluated the capacities of in situ tumor formation of FGD5-AS1-depleted GC cells by subcutaneous xenograft models in nude mice. The results indicated that FGD5-AS1 knockdown significantly inhibited xenograft tumor weight and volume, but had no obvious effect on the body weight of the nude mice (Fig. 4i-l). These results together suggested that knockdown of FGD5-AS1 inhibited GC cell proliferation in vitro and in vivo.

### FGD5-AS1 knockdown induces SASP and DNA damage in GC cells

**Fig. 5:**
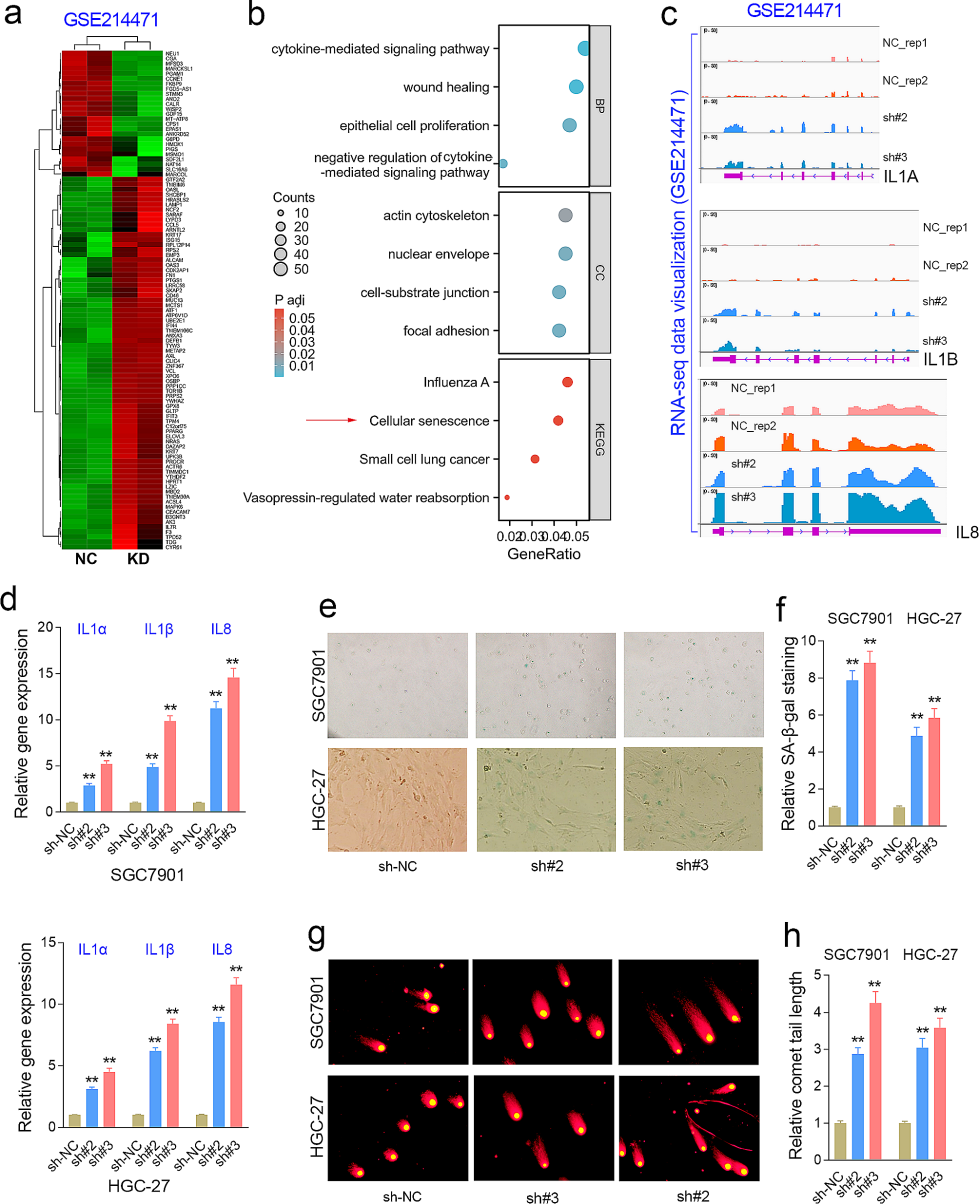
FGD5-AS1 silencing induces senescence and DNA damage in GC cells. (**a**) The RNA-seq analysis was performed in the SGC7901 cell line with or without FGD5-AS1 knockdown. (**b**) The GO/KEGG analysis showed that the differently expressed genes were enriched in cellular senescence signaling. (**c**) The transcript abundance of the SASP factors according to the RNA-seq data of the FGD5-AS1 knockdown. (**d**) The expression levels of cellular senescence biomarkers, including IL1A, IL1B, and IL8/CXCL8, were significantly increased in the FGD5-AS1 depleted GC cell lines. (**e**) The SA-β-gal staining assay showed that FGD5-AS1 knockdown obviously increased the number of senescence cells in the HGC-27 and SGC7901 cell lines. (**f**) The comet assay confirmed that knockdown of FGD5-AS1 significantly promoted DNA damage in the GC cell lines. **, *P* < 0.01

To clarify the underlying mechanism of FGD5-AS1’s pro-tumor effect, RNA sequencing studies (GSE214471) were performed in the SGC7901 cells with or without FGD5-AS1 knockdown. The most significant differentially expressed genes (DEG) after knockdown of FGD5-AS1 were displayed in the heat map (Fig. 5a). The GO/KEGG analysis showed that the DEGs after FGD5-AS1 knockdown were enriched in cellular senescence signaling (Fig. 5b). The RNA-seq analysis and qRT-PCR assays together showed that the SASP-related genes, including IL1A, IL1B, and IL8/CXCL8, were significantly increased in the FGD5-AS1-depleted GC cell lines (Fig. 5c, d). Besides, SA-β-gal staining results showed that FGD5-AS1 knockdown significantly increased the number of senescent GC cells (Fig. 5e, f). Moreover, the comet assays showed that FGD5-AS1 knockdown significantly promoted DNA damage in GC cell lines (Fig. 5g, h).

### FGD5-AS1 is a YBX1-binding lncRNA to stabilize the YBX1 protein

**Fig. 6:**
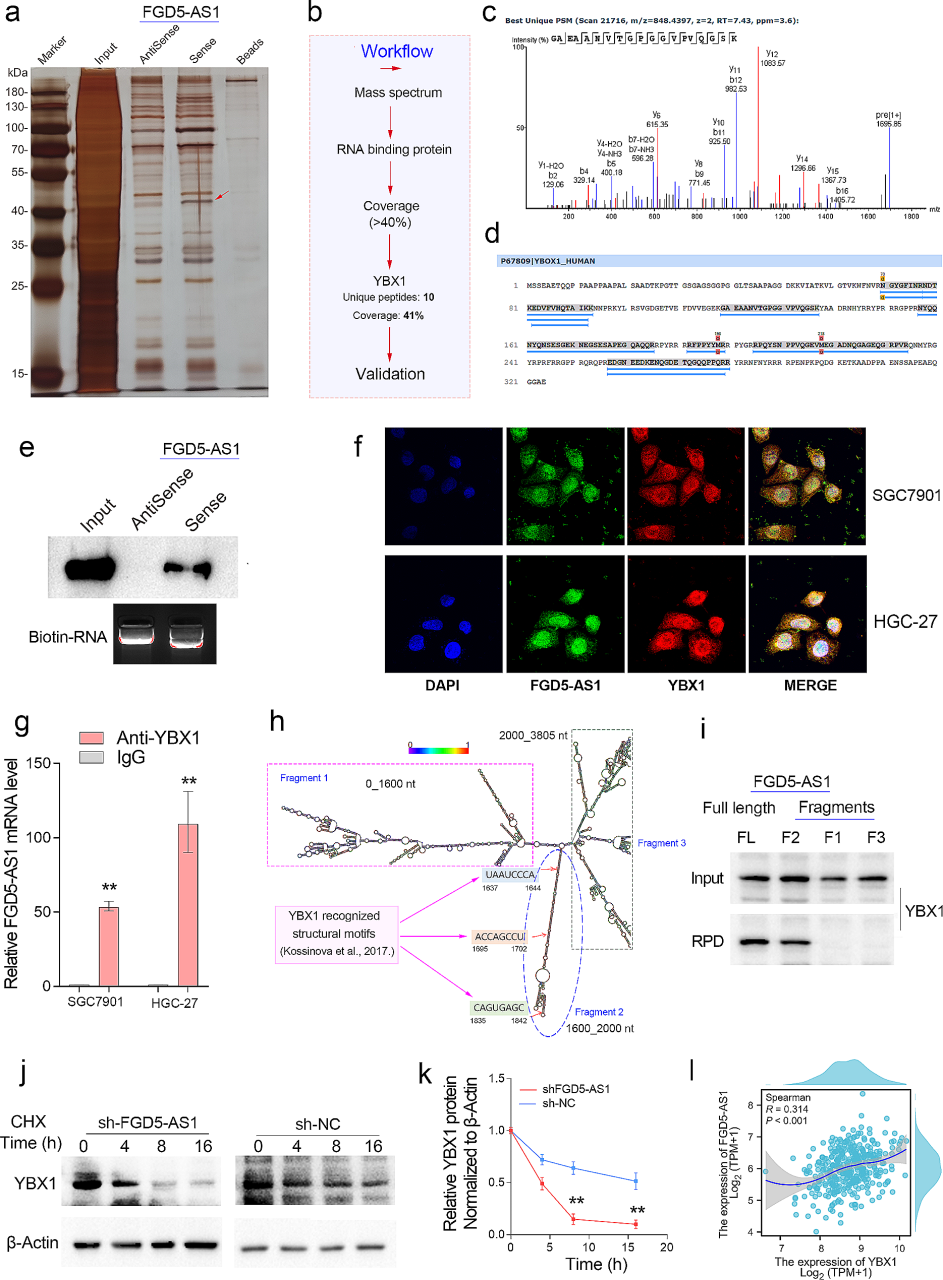
FGD5-AS1 directly binds and stabilizes the YBX1 protein in GC. (**a**) The RNA pull-down assay for identification of proteins interacted with FGD5-AS1 was performed. The sense (S) and anti-sense (AS) of FGD5-AS1 RNA were biotinylated, refolded, and incubated with HGC-27 cell lysates. The red arrow represents the differential band containing the potential FGD5-AS1 binding proteins. (**b**) The differential band was separated for mass spectrum analysis. The FGD5-AS1 binding proteins were finally confirmed according to the workflow. (**c**, **d**) According to the MS analysis, YBX1 was the most possible RNA-binding protein that interacted with FGD5-AS1. (**e**) The western blotting using RNA pulldown samples confirmed the binding of FGD5-AS1 and YBX1. (**f**) FGD5-AS1 has a similar sub-cellular location to the YBX1 protein in the GC cell lines. (**g**) The interaction of FGD5-AS1 and YBX1 was further verified using the RIP-qPCR assay in the GC cell lines. (**h**) The secondary structure of the lncRNA FGD5-AS1 was predicted by the RNAfold web server. According to the predicted structure, we divided FGD5-AS1 into three fragments. Notably, fragment 2 contains three 8-mer linear YBX1 recognized binding motifs. (**i**) The RNA pull-down experiments using three truncated fragments of FGD5-AS1 were performed. It was found that only fragment 2 and full-length FGD5-AS1 fragments could interact with YBX1. (**j**, **k**) The knockdown of FGD5-AS1 significantly prolonged the half-life of the YBX1 protein in the HGC-27 cell line. (**l**) The gene expression correlation analysis showed that FGD5-AS1 was highly co-expressed with YBX1 in GC. **P < 0.01

As a cellular abundant lncRNA, FGD5-AS1’s non-ceRNA function remains largely unknown. Therefore, an RNA pulldown assay was conducted in the HGC-27 cells (Fig. 6a). The conducted mass spectrometry (MS) analysis on the differential band is indicated by the red arrow, and the detail workflow of the MS analysis process is shown in Fig. 6b. According to the MS analysis, the differential protein pulled down by lncRNA FGD5-AS1 is highly likely to be the RNA binding protein YBX1 (Fig. 6b-d). Next, we validated the RNA pulldown results by western blotting using YBX1 antibodies. As expected, the result showed that FGD5-AS1 can directly interact with the YBX1 protein (Fig. 6e). Besides, the fluorescence images captured by laser confocal fluorescence microscopy indicate that the lncRNA FGD5-AS1 and YBX1 proteins are highly co-localized in the HGC-27 cells (Fig. 6f). Consistently, the RNA binding protein immunoprecipitation assay (RIP) showed that the abundance of FGD5-AS1 transcripts pulled down by the YBX1 antibody is tens of times higher than that pulled down by the IgG protein (Fig. 6g).

Next, we are curious about which segment of FGD5-AS1 combines with the YBX1 protein. First, we predicted the secondary structure of lncRNA FGD5-AS1 using the RNAfold web server (http://rna.tbi.univie.ac.at/cgi-bin/RNAWebSuite/RNAfold.cgi). The predicted result is shown in Fig. 6h. Notably, fragment 2 with less than 400 nucleotides contains three adjacent linear 8-mer motifs (UAAUCCCA, ACCAGCCU, and CAGUGAGC) that are reported to be recognized by the YBX1 protein [20]. Thus, we speculated that fragment 2 may be required for the interaction between FGD5-AS1 and YBX1. Given this, FGD5-AS1 transcripts with different truncation lengths were used for RNA pull-down experiments in HGC-27 cells, respectively. As expected, fragment 2 of the FGD5-AS1 alone is sufficient for the binding between FGD5-AS1 and YBX1 (Fig. 6i). Additionally, we further evaluated the effects on FGD5-AS1 or YBX1 expression by the interaction of FGD5-AS1 and YBX1 after knockdown of either FGD5-AS1 or YBX1 in the HGC-27 cells. The results showed that knockdown of FGD5-AS1 significantly prolonged the half-life of the YBX1 protein in the HGC-27 cells (Fig. 6j, k). However, knockdown of YBX1 had no obvious effect on the expression of FGD5-AS1 in the GC cell lines HGC-27 and SGC7901 (Fig S2a-c). Consistently, expression correlation analysis showed that FGD5-AS1 was highly co-expressed with YBX1 in GC (Fig. 6l). These results implied that FGD5-AS1 directly binds and stabilizes the YBX1 protein in GC.

## FGD5-AS1 depletion inhibits GC cell proliferation and induces SASP via YBX1

**Fig. 7:**
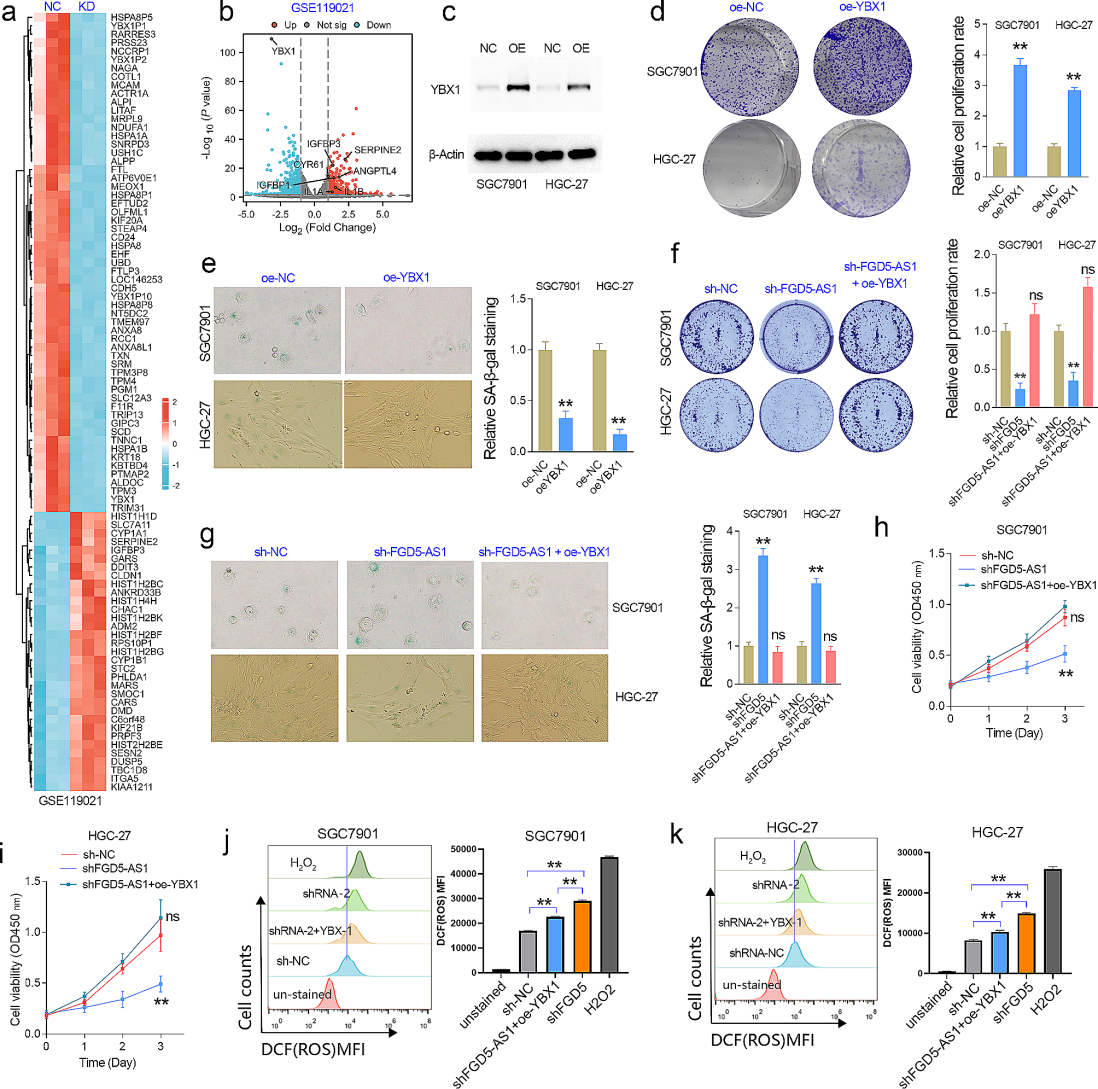
FGD5-AS1 promoted GC proliferation and repressed senescence in a YBX1-dependent manner. (**a**) The differentially expressed genes after YBX1 knockdown were shown in the heat map after RNA-seq analysis. (**b**) YBX1 knockdown significantly increased the expression level of SASP factors, including IL1A, IL1B, IGFBP1/3, CYR61, ANGPTL4, and SERPINE2 in the GC cell line BGC823. (**c**) The western blotting assays showed that YBX1 was successfully overexpressed in GC cell lines. (**d**) The cell colony formation assay was performed in the GC cell lines with or without YBX1 overexpression. (**e**) The SA-β-gal staining assay showed that YBX1 overexpression significantly inhibited cell senescence in the HGC-27 and SGC7901 cell lines. (**f**) The rescue colony formation assay showed that the inhibitory effects of FGD5-AS1 depletion on cell proliferation could be rescued by additional YBX1 overexpression in GC. (**g**) The rescue SA-β-gal staining assay showed that the promotion effects of FGD5-AS1 knockdown on cell senescence could be rescued by additional YBX1 overexpression in GC. (**h**, **i**) The rescue CCK-8 assay showed that the inhibitory effects of FGD5-AS1 knockdown on GC cell growth could be rescued by additional YBX1 overexpression. (**j**, **k**) Evaluate the effect of FGD5-AS1 knockdown on intracellular ROS content using flow cytometry. The 2’,7’-dichlorofluorescein (DCF) is an oxidatively sensitive fluorescent probe used to measure intracellular reactive oxygen species (ROS) production. The inhibitory effects of FGD5-AS1 knockdown on ROS production of GC cell lines could be rescued by additional YBX1 overexpression. **P < 0.01

Previous studies have implied that YBX1 plays an inhibitory role in cell senescence or aging [21–23]. Zhang and colleagues have reported the interaction between the lncRNA HOXC-AS3 and the YBX1 protein in GC [24]. During this study, they uploaded the RNA-seq data (GSE119021) of YBX1 knockdown in the GC cell line BGC-823. After RNA-seq analysis of the GSE119021 dataset, we found that YBX1 knockdown significantly increased several SASP-related genes, such as IL1A, IL1B, IGFBP1/3, CYR61, SERPINE2, and ANGPTL4 in BGC-823 cells (Fig. 7a, b). These results indicated that YBX1 may function as a repressor in GC cell senescence. To validate this speculation, we first evaluated the effect of YBX1 overexpression on GC cell proliferation and senescence in the HGC-27 and SGC7901 cell lines. The CCK-8 and SA-β-gal staining assays showed that YBX1 overexpression significantly increased GC cell proliferation abilities, but inhibited the degree of aging of GC cells (Fig. 7c-e). Besides, YBX1 overexpression significantly decreased the expression of SASP-related factors (Fig S2d-f). Thus, we speculated that FGD5-AS1 may regulate GC cell proliferation and senescence via YBX1. To verify this possibility, the rescue assays were conducted on GC cell lines. As expected, the inhibitory effect of FGD5-AS1 depletion on GC cell proliferation or the promoting effect of FGD5-AS1 depletion on aging of GC cells can be at least partially restored by overexpression of YBX1 (Fig. 7g-j), indicating that FGD5-AS1 regulates cell proliferation and aging in a YBX1 dependent manner.

A previous study has reported that YBX1 plays a profound role in inhibiting ROS generation by interacting with Kindlin-2 in renal cell carcinoma [25]. Increasing studies have indicated that reactive oxygen species (ROS) is one of the important factors leading to aging [26]. Thus, we evaluated the role of FGD5-AS1 and YBX1 on the ROS production of GC cell lines by flow cytometry. Additionally, the promotion effect of FGD5-AS1 knockdown on ROS production can be rescued by additional YBX1 overexpression in GC cells (Fig. 7j, k). These results showed that FGD5-AS1 promotes GC cell senescence, proliferation, and ROS generation in a YBX1-dependent manner.

## Discussion

Metastasis is the leading cause of cancer-related deaths. The epithelial-mesenchymal transition (EMT) process endows tightly anchored epithelial cells with invasive ability, and is therefore considered a crucial step in the early stage of cancer metastasis [27]. Accordingly, we previously found that the degree of EMT progression is closely related to the poor prognosis in GC [27]. Likewise, Cristescu et al. divided GC patients into four subtypes (MSS/TP5−, MSS/TP53+, MSI and MSS/EMT), and found that the EMT subtype of GC patients possessed the worst survival [19]. As a master regulator of cellular EMT signaling, transcription factor ZEB1 is recognized as a core driver of tumor metastasis [27]. Accumulating evidence has shown that ZEB1 plays an oncogenic role in GC by driving metastasis [28, 29].

LncRNA has been proven to be widely and profoundly involved in almost all processes of tumor occurrence and development. Currently, there are few reports on which lncRNAs are regulated by ZEB1. In this study, we chose ZEB1 as the entry point to attempt to screen for lncRNAs associated with GC metastasis using the high-throughput RNA sequencing method. As mentioned earlier, the widespread low-abundance expression of lncRNA inevitably limits its real biological function in cellular events [8]. Thus, although a total of 24 lncRNAs were identified to be significantly regulated by the EMT-TF ZEB1, we preferably selected FGD5-AS1, a cellular high-abundance lncRNA, to conduct follow-up studies.

In the present study, we identified FGD5-AS1 as a direct downstream target lncRNA of the EMT-TF ZEB1 in GC. Therefore, molecular typing studies have shown that FGD5-AS1 is an EMT-related lncRNA that is highly co-expressed with mesenchymal markers, including ZEB1. Besides, FGD5-AS1 overexpression was clinically associated with metastasis and poor survival in GC. And the loss-of-function studies revealed that FGD5-AS1 depletion inhibited GC proliferation in vitro and in vivo, suggesting FGD5-AS1 was a tumor driver gene in GC. Mechanistically, FGD5-AS1 promoted GC progression by repressing cell senescence in a YBX1-dependent manner. Our finding highlights that targeting the FGD5-AS1/YBX1 axis might be a promising anti-cancer strategy for GC.

Unlike the well-known nucleus-localized cellular high-abundant lncRNAs (such as MALAT1, NORAD, and NEAT1), lncRNA FGD5-AS1 was distributed in both the cytoplasm and the nucleus. Perhaps because FGD5-AS1 can be localized in the cytoplasm, almost all retrieved studies on FGD5-AS1 are based on the ceRNA mechanism to reveal its function in tumors. For example, Gao et al. and Feng et al. have reported that lncRNA FGD5-AS1 promotes GC progression by sponging miR-153-3p and miR-195 [30, 31]. However, little is known about the non-ceRNA functions of FGD5-AS1 in cancer.

Herein, we identified FGD5-AS1 as a YBX1-binding lncRNA, since its mRNA contains three 8-mer linear YBX1-recognized motifs (UAAUCCCA, ACCAGCCU, and CAGUGAGC). And the interaction between FGD5-AS1 and YBX1 significantly prolonged the half-life of the YBX1 protein (Fig. 6j, k), but had no significant effect on the mRNA stability of FGD5-AS1 in GC cells. The RNA binding protein YBX1 has been reported to be an oncogenic driver in GC [32–34]. Currently, many studies have reported that non-coding RNAs could drive GC through interaction with YBX1 [24, 35, 36]. Furthermore, our rescue assays confirmed that FGD5-AS1 promoted GC progression in a YBX1-dependent manner. In addition, a recent study reported that YBX1 negatively regulates cell PANoptosis, but positively regulates chemoresistance and tumor progression in GC [37]. Consistently, FGD5-AS1 knockdown also significantly induces cell PANoptosis and inhibits the chemoresistance of GC cells. These findings highlight that targeting the FGD5-AS1/YBX1 axis might be a promising anti-cancer strategy for GC.

It has been reported that YBX1 plays an inhibitory role in cell senescence or aging [21–23]. Cui and colleagues found that YBX1 can interact with Kindlin-2 to repress ROS generation in renal cell carcinoma [25]. In this work, we also found that FGD5-AS1 depletion significantly induces the expression of SASP-related factors and ROS generation in GC cells. And the promotion effect of cell senescence and ROS generation of FGD5-AS1 knockdown could be restored by additional YBX1 overexpression, suggesting FGD5-AS1 regulates cell senescence and ROS generation in a YBX1-dependent manner. Cellular senescence is a stable, growth arrest terminal state that is typically induced by stress signals, such as DNA damage, oncogene activation, oxidative stress, or exposure to exogenous toxins [38]. Consistently, FGD5-AS1 knockdown significantly promoted DNA damage and ROS production in GC cells, thereby driving GC cell senescence. Many studies have found that cellular aging plays a “braking” role in the occurrence and progression of cancer [39]. Senescence causes tumor cells to enter a stable state of cell cycle arrest, thereby becoming a natural barrier to inhibit tumor progression and metastasis [40]. Therefore, we considered that FGD5-AS1 promotes GC progression by repressing cell senescence and ROS production via binding and stabilizing the YBX1 protein.

In addition, antisense lncRNAs tend to be highly co-expressed with the corresponding parental genes and adjacent genes [41–44]. Herein, we also found that FGD5-AS1 was highly co-expressed with multiple adjacent genes located at chromosomes 3p25.1 and 3p21.31, including NR2C2, CAPN7, RBSN, and so on (Figs S3 a-e). Interestingly, knockdown of FGD5-AS1 slightly decreased the expression of NR2C2, CAPN7 and RBSN in GC cell lines (Fig S3f). Nevertheless, the molecular mechanism underlying the co-expression between FGD5-AS1 and adjacent genes remains to be further elucidated.

**Fig. 8:**
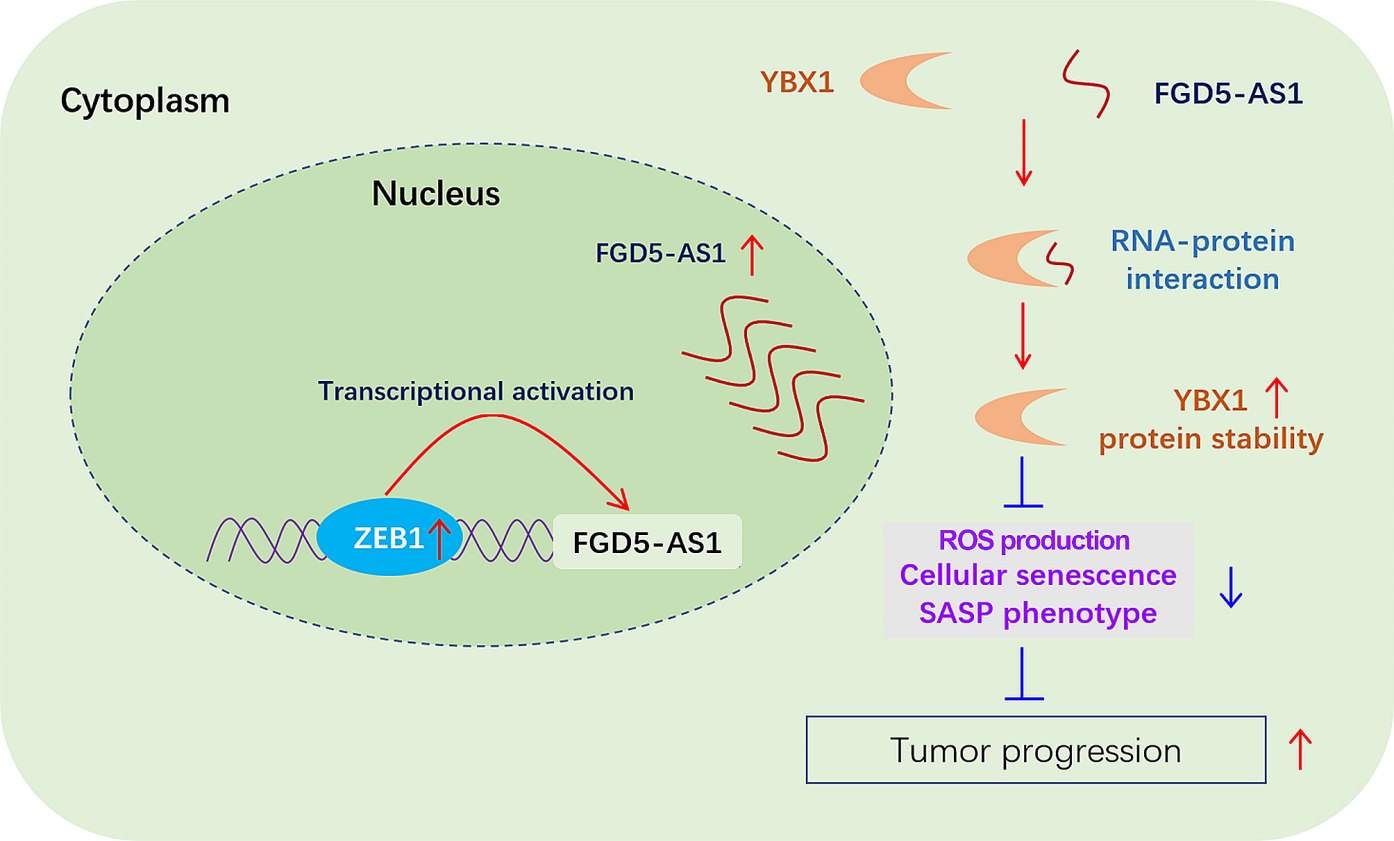
The working model of the role of lncRNA FGD5-AS1 in driving GC progression. The cellular high-abundant lncRNA FGD5-AS1 was transcriptionally activated by the EMT-TF ZEB1 in GC. Due to the upregulation of ZEB1, lncRNA FGD5-AS1 expression was increased, and its overexpression was clinically associated with metastasis and poor survival in GC. Mechanically, FGD5-AS1 directly binds to the YBX1 protein to enhance its protein stability. FGD5-AS1 promotes GC progression by repressing cell senescence and ROS production in a YBX1-dependent manner

In summary, FGD5-AS1 is a cellular high-abundant lncRNA that is transcriptionally activated by ZEB1. Our finding highlights that lncRNA FGD5-AS1 promotes GC progression by inhibiting cell senescence and ROS production through binding and stabilizing the YBX1 protein (Fig. 8).

## Materials and methods

### Bioinformatics analysis

The RNA-seq data of the Cancer Genome Atlas (TCGA) stomach cancer cohort was downloaded from the cBiopPortal web server. The survival analysis of the TCGA_STAD cohort was conducted using the GEPIA web tool. The global gene expression data of the GSE62254 dataset was downloaded from the Gene Expression Omnibus (GEO) on the NCBI web server. The clinical information of the ACRG cohort (GSE62254) was downloaded from the supplementary data of Cristescu [19]. The gene expression data and clinical information of the Australian GC cohort (GSE35809) was download from the GEO database. The DEseq2 R package was used to identify the significant differentially expressed genes (DEGs) of the RNA-seq databases (GSE214471 and GSE119021). The chip-seq data (GSE32465) using ZEB1 antibodies in the HepG2 cell line was visualized in the Cistrome web tool. The RNA secondary structure of FGD5-AS1 was performed using the RNAfold web server. The expression correlation analysis in this study was conducted using the GEPIA web tool.

### Molecular typing analysis

The transcriptome data of the normal stomach tissues from the GTEx cohort was obtained as we previously described [4]. The unsupervised clustering analysis was performed based on the expression pattern of classic EMT-related biomarkers. The clustering method is complete linkage, and the distance measurement method is Pearson. Patients are divided into two subgroups according to their distinct EMT signatures.

### Cell transfection and establishment of cell lines

The GC cell lines (AGS, HGC-27, and SGC7901) and the normal gastric cell line GES-1 were purchased from the Shanghai Cell Bank of the Chinese Academy of Sciences. The cells were cultured in DMEM medium containing 10% fetal bovine serum (FBS) at 37 ^o^C in 5% CO_2_. For cell transfection, GC cell lines were seeded into 6-well plates and grown overnight. The next day, when the cell plating density reached 20–30%, cells were transfected with siRNAs (final concentration, 50 nM) by Lipofectamine 2000 (Invitrogen) according to the manufacturer’s instructions. The siRNAs (Genepharma, Shanghai, China) used in this study are listed in Table S1. For stable knockdown of FGD5-AS1, the lentiviruses of shFGD5-AS1 were purchased from Genepharma. Lentiviral transfection was performed according to the manufacturer’s instructions. At the indicated time points, the cells were harvested for mRNA and protein analysis as well as for other assays.

### Clinical GC samples

The study protocol was approved by the Human Research Ethics Committee of Hubei University of Medicine. The procedures were in accordance with the Helsinki Declaration of 1975. Written informed consent was obtained from all patients. Tissue samples were immediately frozen in liquid nitrogen after resection and stored at − 80 °C until use. All samples were pathologically confirmed.

### RNA isolation and quantitative RT-PCR

The qPCR analysis was conducted using One Step ^TB^ Green PrimeScript^™^ RT-PCR Kit II (Takara) as previously described [43]. The ACTB was used as reference gene (qF: ATCGTCCACCGCAAATGCTTCTA; qR: AGCCATGCCAATCTCATCTTGTT). Each gene was run in triplicate. Relative fold changes in gene expression were calculated using the comparative ^ΔΔ^Ct method. The primer sequences for other genes in this study are as follows (indicated as 5′ → 3′): ZEB1_qF: CCTCTTCACAGGTTGCTCCT; ZEB1_qR: TGCAGGAGCTGAGAGTCA; YBX1-qF: TCGCCAAAGACAGCCTAGAGA; YBX1-qR: TCTGCGTCGGTAATTGAAGTTG; MALAT1-qF: AAAGCAAGGTCTCCCCACAAG MALAT1-qR: GGTCTGTGCTAGATCAAAAGGCA; FGD5-AS1-qF: CCTTGTCCTTCCCTGTTTCA; FGD5-AS1-qR: ACTGGGCACTTGATGCTTTC; IL1A-qF: ACCTCACGGCTGCTGCATTACA; IL1A-qR: TCCTTCAGCAGCACTGGTTGGT; YBX1-qF: CAGCCTTCCCAGCTATTCAG; YBX1-qR: ATAGGACAGTGCCCCACATC; IL6-qF: GCACTGGCAGAAAACAACCT; IL6-qR: CAGGGGTGGTTATTGCATCT; IL1B-qF: TTGTTGAGCCAGGCCTCTCT; IL1B-qR: CCAAATGTGGCCGTGGTT; IL8-qF: CCAGGAAGAAACCACCGGAA; IL8-qR: CTCCTTGGCAAAACTGCACC.

### RNA sequencing

The RNA-seq studies were conducted in the FGD5-AS1 depleted SGC7901 cells. Briefly, a total amount of 1.5 µg RNA per sample was used as input material for the RNA sample preparations. The whole process of library construction and sequencing was performed at Shanghai Lifegenes Technology Co., Ltd. The RNA-seq data was uploaded to the GEO section of the NCBI web server. The GEO accession number was GSE214471.

### Mouse xenograft model

The study protocol was approved by the Experimental Animal Research Ethics Committee of Hubei University of Medicine. All animals were treated in accordance with the guidelines of the Committee on Animals of the Hubei University of Medicine. HGC-27 cells (shNC and shFGD5-AS1) were injected into the subcutaneous tissue of female BALB/c nude mice. After 28 days, all the mice were euthanized, and the tumors were collected for weighing and volume measurement. The tumor volume was calculated using the following formula: volume = length × (width)^2^/2.

### Chromatin immunoprecipitation assay

Briefly, the AGS cells were transfected with ZEB1_3*FLAG overexpression plasmids (pCDNA 3.1 vector) using Lipo 2000 (Promega, USA). Then, all cells were collected and fixed for 10 min at 37 with 1% formaldehyde, followed in sequence with SDS lysis and DNA shearing, protein and DNA immunoprecipitation, cross-linked DNA reversal, and DNA purification. The DNA fragments immunoprecipitated by magnetic beads-conjugated mouse anti-DDDDK-Tag mAb (AE037, Proteintech, Wuhan, China) or IgG (negative control) were detected by qPCR assays. The primer sequences for chip assay are listed as follows: FGD5-AS1_Chip-qF: ATCGTGGACTCGAAATGCTT; FGD5-AS1_Chip-qR: CGGAAGAGGGTGGTCCTTA; Ubiquitin_Chip-qF: TGGGTCCGATTATTGAATGG; Ubiquitin_Chip-qR: AGCTGGGTGTCCAGGTTAAA.

### RNA immunoprecipitation assay

The RNA immunoprecipitation assay was conducted as we previously described [42]. Briefly, GC cell lines SGC7901 and HGC-27 were harvested and lysed in RIP lysis buffer according to the manufacturer’s protocol. Protein A/G beads were added for a further 4 h incubation with cell lysates at room temperature. After the beads were washed, immunoprecipitated RNAs were extracted and purified for qRT-PCR analysis. The normal rabbit IgG was used as the negative control group.

### RNA pull-down assay

The RNA pull-down assay was conducted as we previously described [43]. Briefly, the full-length of sense and antisense FGD5-AS1 were cloned into pGEM-T Easy vector (Promega) for in vitro transcription. The generated RNA was labeled with biotin at the 3′ end. The HGC-27 cells were harvested and lysed according to the manufacturer’s protocol (Thermo Scientific, Cat No. 74,830). The sense and antisense RNAs were incubated with cell lysates overnight at 4 °C. The RNA-binding protein complexes were washed five times with ice-cold wash buffer and boiled in SDS lysis buffer for western blot assay and SDS-PAGE analysis.

### Cell senescence detection

The SA-β-gal staining was conducted using Senescence β-Galactosidase Staining Kit (C0602, Beyotime, Shanghai, China) according to the manufacturer’s protocol. Briefly, GC cell lines with or without FGD5-AS1 knockdown (or YBX1 overexpression) were cultured for 3 days in the 6-well plates. Then, cells were washed in PBS, fixed with 3% formaldehyde, and incubated with SA-β-Gal staining solution at 37 °C for 4 h. The stained cells were washed with water and examined with a microscope.

#### Comet experiments

The GC cell lines with or without FGD5-AS1 knockdown were seeded in the 6-well plates. After the first layer of 1% normal melting point agarose gel was prepared with glass slides, the collected cells were mixed with 0.7% low melting point agarose gel and laid on the first layer of gel to prepare the second layer of gel. Then PBS was cracked at 4 °C for 2 h and rinsed for 3 min. The slides were soaked in alkaline electrophoresis buffer to unhelix for 30 min. The electrophoresis was then turned on for 30 min. After electrophoresis, the slides were placed in a neutral buffer for 10 min. Finally, the propidium-lodide solution was added to the slide, incubated in the dark for 20 min, and then observed under a fluorescence microscope.

### ROS detection

The relative ROS production level was determined using flow cytometry. The levels of hydrogen peroxide in GC cells were determined by staining the cells with a 2’,7’-Dichlorofluorescein (DCF) probe. The intensity of DCF fluorescence is proportional to the amount of peroxide produced in the cells. Briefly, GC cells were incubated with DCF at 37 °C for 15 min. Then, cells were removed, washed, and resuspended in PBS and analyzed for DCF fluorescence by using the CytoFLEX machine (Beckman, USA). The hydrogen peroxide treatment was used as a positive control group.

#### Western blot assay

The immunoblotting assays were performed as we described previously [45]. Briefly, the GC cell lines were lysed in RIPA buffer with 1 mM PMSF. The protein concentration of all samples was quantified and normalized using BSA method. Then, the protein samples were electrophoresed and transferred to the PVDF membranes. After incubating with primary antibodies and secondary antibodies, the final images were visualized using a Bio-Rad Imaging System (USA). The primary antibodies used in this study were as follows: ZEB1 (21544-1-AP, Proteintech, Wuhan, China), YBX1 (A6799, ABclonal, Wuhan, China), and β-Actin (AC043, ABclonal, Wuhan, China).

### Statistical analysis

For gene expression analysis of different subtypes of GC, the P values were estimated using Mann–Whitney nonparametric test. Survival curves were calculated using the Kaplan-Meier method, and differences between the curves were analyzed using the log-rank test. All the rest of the experiments used an unpaired t-test or a one-way ANOVA test. For all experiments, a minimum of triplicates per group and repetition at least three times were applied to achieve reproducibility. All tests with p values less than 0.05 are considered statistically significant.

### Electronic supplementary material

Below is the link to the electronic supplementary material.

Supplementary Material 1

## Data Availability

The datasets generated during the current study are available in this article or in supplementary files.
