# Supplementary material for: lncRNA FGD5-AS1 is required for gastric cancer proliferation by inhibiting cell senescence and ROS production via stabilizing YBX1
Source: J Exp Clin Cancer Res. 2024 Jul 5;43:188. doi: 10.1186/s13046-024-03103-x (PMC11225384; doi:10.1186/s13046-024-03103-x)

**Supplemental information**

**Figure S1** The clinical value of ZEB1 overexpression was analyzed in GC. (a) Gene expression analysis in the GSE122401 cohort showed that ZEB1 expression was significantly upregulated in GC. (b, c) ZEB1 overexpression was positively associated with the degree of the T-stage or N-stage of GC patients in the GSE62254 cohort. (d) The expression level of ZEB1 is positively correlated with the number of positive lymph nodes in the GSE62254 cohort. (e) ZEB1 overexpression predicted a poor overall prognosis in the GSE62254 cohort. (f, g) ZEB1 overexpression was positively associated with the degree of the T-stage or N-stage of GC patients in the TCGA_STAD cohort. (h) The expression level of ZEB1 is positively correlated with the number of positive lymph nodes in the TCGA_STAD cohort. (i) The GC patients with high ZEB1 expression possessed poor overall survival in the TCGA_STAD cohort. ****, P < 0.0001; ***, P < 0.001; **, P < 0.01; *, P < 0.05


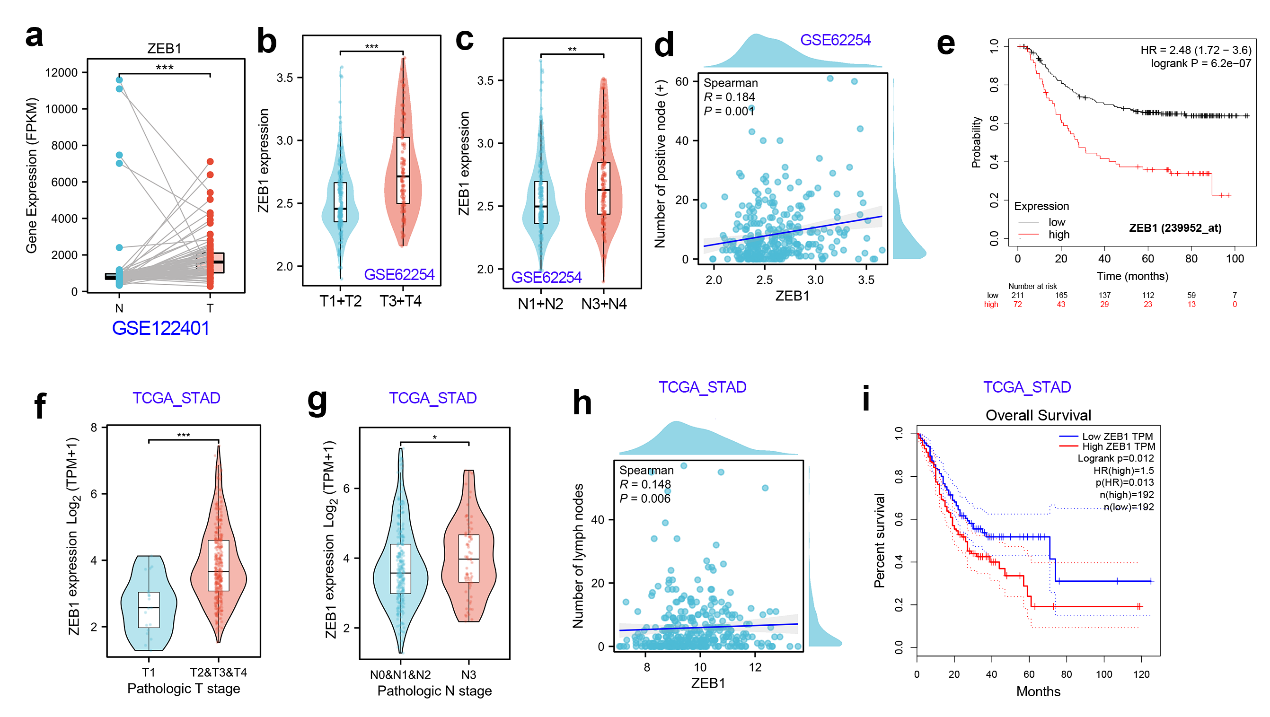


**Figure S2** YBX1 cannot regulate FGD5-AS1 expression but inhibits the expression of SASP-related factors. (a) RNA-seq analysis of the GSE119201 dataset showed that YBX1 knockdown had no significant effect on the expression level of FGD5-AS1 in the GC cell line BGC823. (b) The overexpression efficiency of YBX1 in GC cell lines was determined by qRT-PCR analysis. (c) YBX1 overexpression has no obvious effect on the expression of GFD5-AS1 in GC cell lines. (d-f) YBX1 overexpression significantly reduced the expression of SASP-related factors, including IL1A, IL1B, and IL8, in GC cell lines.


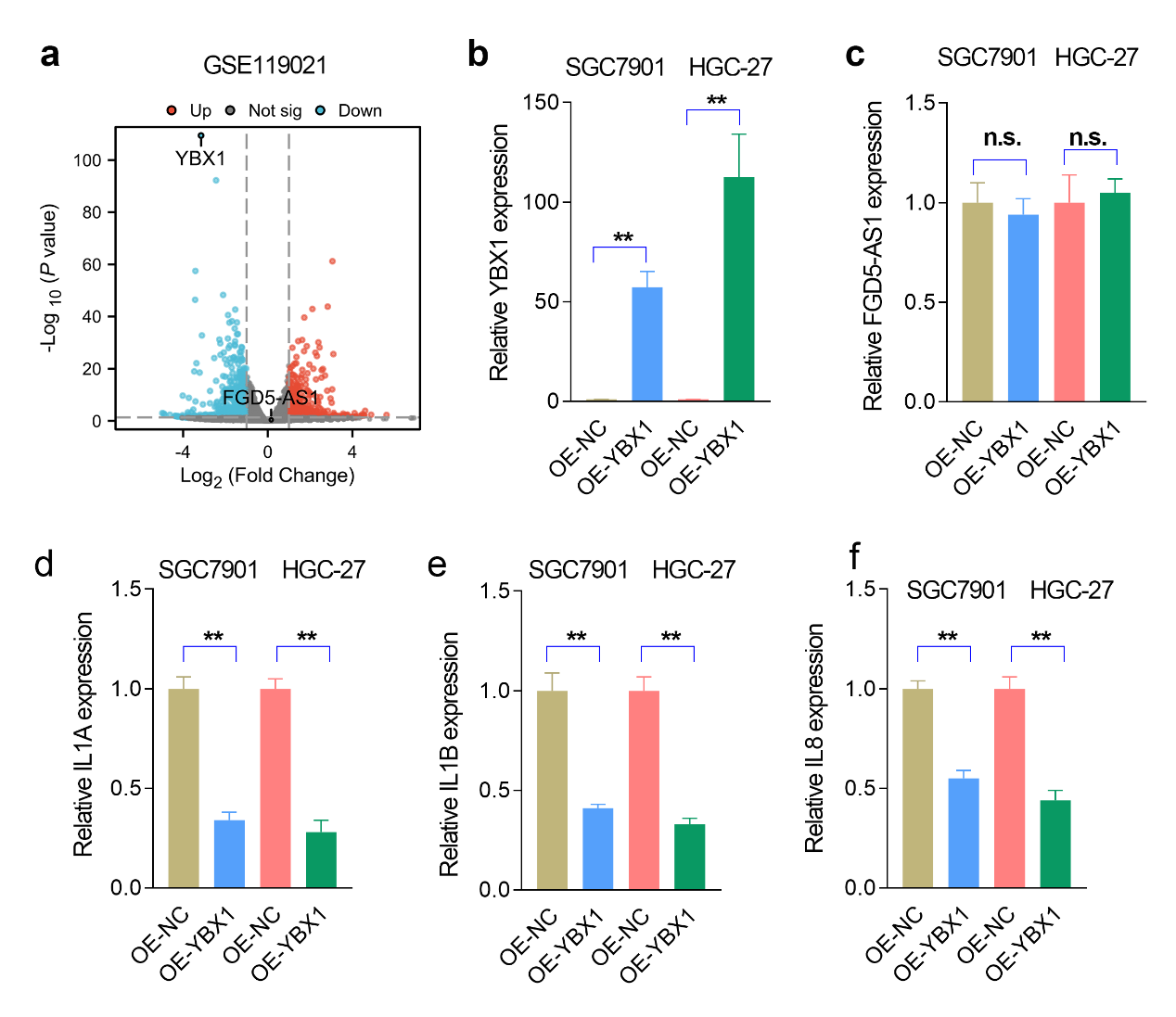


**Figure S3** FGD5-AS1 was highly co-expressed with adjacent genes in pan-tissue and pan-cancer. (a) The gene expression correlation analysis between FGD5-AS1 and other genes was conducted in the TCGA_SATD cohort. NR2C2 is one of the most significantly co-expressed genes with FGD5-AS1. (b) The heat map displayed the top 20 genes most significantly co-expressed with FGD5-AS1. Most of them were located on chromosome 3. (c) NR2C2, RBSN, and CAPN7 were the three genes closest to the physical location of FGD5-AS1. (d, e) NR2C2, RBSN, and CAPN7 were highly co-expressed with FGD5-AS1 in pan-tissue and pan-cancer. (f) Knockdown of FGD5-AS1 significantly decreased the expression of CAPN7, NR2C2, and RBSN in GC cell lines. **, P < 0.01


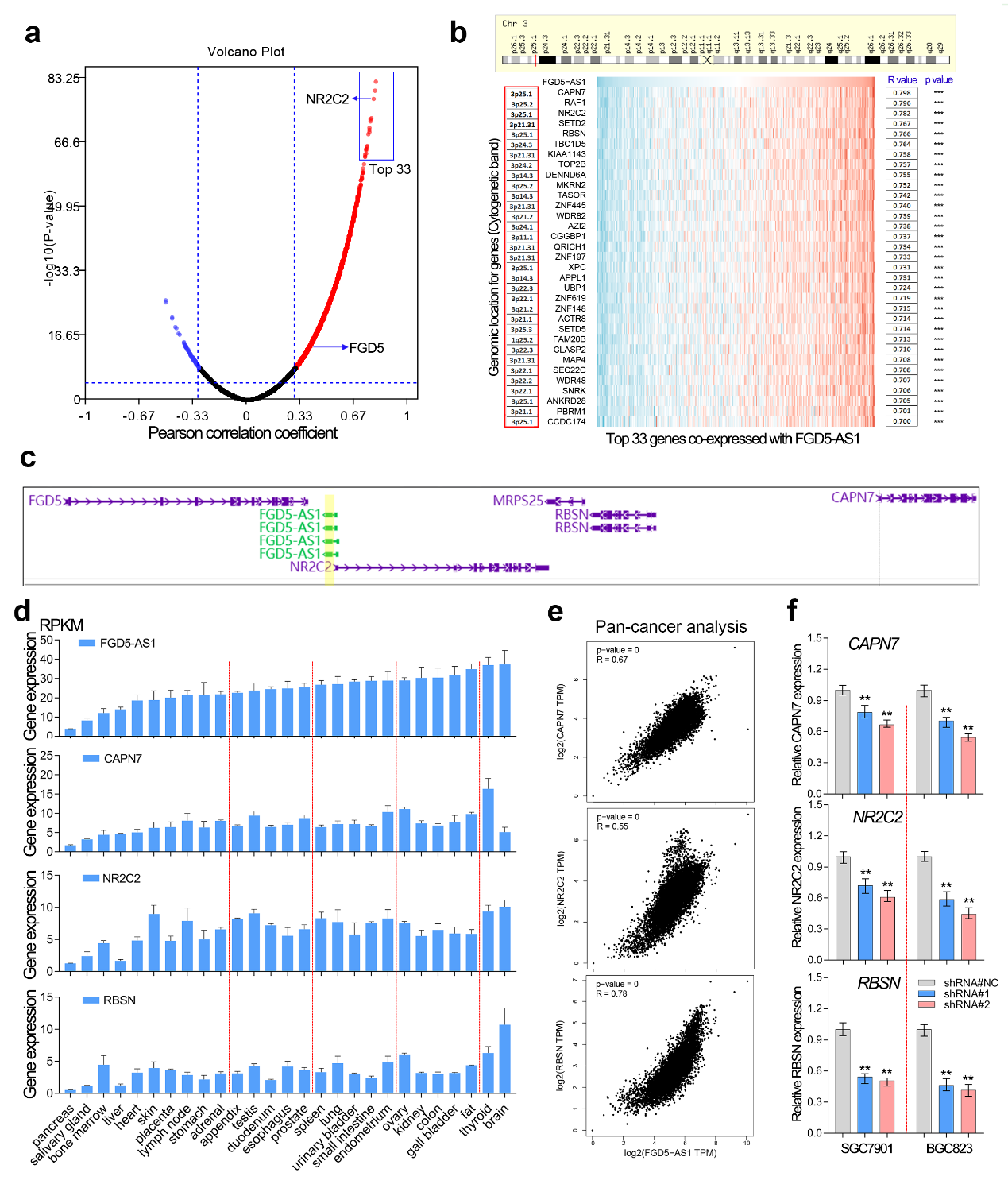

Supplement: Supplementary file 1 — Supplementary Material 1 [file 13046_2024_3103_MOESM1_ESM.docx]
